# Local excision and treatment of early node-negative anal squamous cell carcinomas in a highly HIV prevalent population

**DOI:** 10.1007/s10151-021-02473-0

**Published:** 2021-06-12

**Authors:** D. R. L. Brogden, C. Kontovounisios, I. Chong, D. Tait, O. J. Warren, M. Bower, P. Tekkis, S. C. Mills

**Affiliations:** 1 Chelsea and Westminster Hospitals NHS Foundation Trust, London, UK; 2 Imperial College London, London, UK; 3 Royal Marsden NHS Foundation Trust, London, UK; 4 Institute of Cancer Research, London, UK

**Keywords:** Anal squamous cell carcinoma, HIV, Chemoradiotherapy, HPV

## Abstract

**Background:**

Anal squamous cell carcinoma (ASCC) is an uncommon cancer associated with human immunodeficiency virus (HIV) infection. There has been increasing interest in providing organ-sparing treatment in small node-negative ASCC’s, however, there is a paucity of evidence about the use of local excision alone in people living with HIV (PLWH). The aim of this study was to evaluate the efficacy of local excision alone in this patient population.

**Methods:**

We present a case series of stage 1 and stage 2 ASCC in PLWH and HIV negative patients. Data were extracted from a 20-year retrospective cohort study analysing the treatment and outcomes of patients with primary ASCC in a cohort with a high prevalence of HIV.

**Results:**

Ninety-four patients were included in the analysis. Fifty-seven (61%) were PLWH. Thirty-five (37%) patients received local excision alone as treatment for ASCC, they were more likely to be younger (*p *= 0.037, ANOVA) and have either foci of malignancy or well-differentiated tumours on histology (*p *= 0.002, Fisher’s exact test).

There was no statistically significant difference in 5-year disease-free survival and recurrence between treatment groups, however, patients who had local excision alone and PLWH were both more likely to recur later compared to patients who received other treatments for ASCC. (72.3 months vs 27.3 months, *p *= 0.06, ANOVA, and 72.3 months vs 31.8 months, *p *= 0.035, ANOVA, respectively).

**Conclusions:**

We recommend that local excision be considered the sole treatment for stage 1 node-negative tumours that have clear margins and advantageous histology regardless of HIV status. However, PLWH who have local excision alone must have access to an expert long-term surveillance programme after treatment to identify late recurrences.

## Introduction

Anal squamous cell carcinoma (ASCC) is an uncommon cancer that accounts for 1–2% of gastrointestinal malignancies [2]. Its worldwide incidence is 1–2 cases per 100,000 people per year [3].

The incidence of ASCC is rapidly increasing worldwide [4]. A common risk factor of ASCC is human immunodeficiency virus (HIV), as people living with HIV (PLWH) are living now longer on advanced antiretroviral therapies there is also an associated rise in incidence of ASCC in PLWH and men who have sex with men (MSM).

ASCC has a dysplastic precursor; high-grade squamous interepithelial lesion (HSIL) which is related to persistent oncogenic human papillomavirus (HPV) infection. HSIL can progress to ASCC, however, there is much discrepancy in the literature regarding the progression rates [5–8].

It is believed, that like cervical cancer, a screening programme for HSIL could prevent the progression of HSIL to ASCC. As yet, there is little consensus on the relevance of HSIL screening for the prevention of ASCC as there is insufficient evidence available of its efficacy in preventing ASCC [9–14]. Most current clinical guidelines recommend the surveillance of high-risk populations, in particular HIV-positive MSM, but there is an insufficient evidence base to propose a method or schedule of best practice to do so.

One of the suggested benefits of a successful screening programme in high-risk individuals would be the early detection of a cancer to allow for an organ sparing approach to treatment. This would be of significant quality of life benefit to PLWH as they often develop ASCC at a younger age [15] and treatment to the perianal region has a significant impact on the sexual and psychological wellbeing of MSM.

As a result, there is increasing interest in undertaking local excision alone for early-stage ASCC tumours without nodal involvement. Indeed, the PLATO trial (SRCTN88455282) has recently begun; its ACT3 arm is a non-randomised study comparing the outcomes of patients with fully excised T1 perianal ASCC’s (< 1 mm) who will have local excision as their only treatment to patients with T1 perianal ASCC excised with inadequate margins (> 1 mm) who will also receive chemoradiotherapy. [16]. The trial results are not expected in the near future; however, in the meantime, other findings on the management of early ASCC have been published. [17] used the American National Cancer Database to compare the outcomes of 2243 stage 1 tumours after local excision only or chemoradiotherapy [17] and [18] interrogated the Surveillance, Epidemiology and End Results (SEER) database to investigate the outcomes of stage 1 tumours and the outcomes after different treatment modalities [18]. Where other smaller local case series have also been presented [19–22], none so far have identified any difference in survival, suggesting that the treatment of early ASCC tumours may be successfully limited to local excision alone in selected patients. However, none of the studies in the literature outside our clinical centre [23] have looked specifically at the benefits of local excision alone in PLWH. This is a significant gap in the literature as this patient group are the most likely to achieve early detection due to HSIL surveillance programmes and also as they are more likely to have a later recurrence [15] their long-term outcomes after organ sparing treatment may not be as advantageous.

We present a 20-year case series of the treatment of early-stage node-negative (1, 2A and 2B) ASCC tumours in a tertiary referral centre for HIV where there is a high prevalence of PLWH in our general practice. Our clinical centre also benefits from the availability of a screening programme for PLWH with particular emphasis on PLWH MSM.

## Materials and methods

We undertook a case series review of all early-stage cancers within a retrospective cohort study completed at our clinical centre in a prospectively collected database. We followed the strengthening the reporting of observational studies in epidemiology (STROBE)” statement in designing and undertaking the cohort study [24]. Ethical approval was granted from the London-Westminster Research and Ethics Committee prior to commencing the cohort study.

### Inclusion criteria for case series

**Fig. 1:**
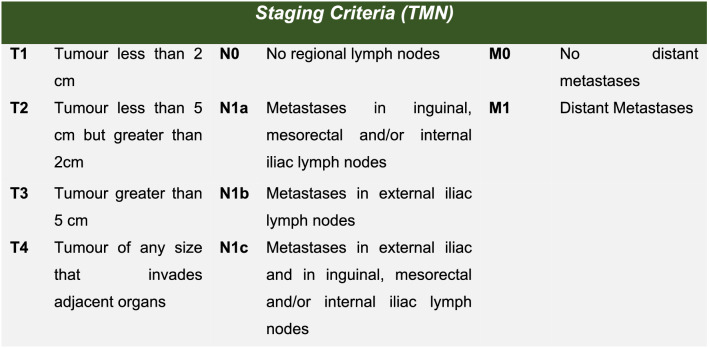
American Joint Committee on Cancer recommended TMN staging 8th Edition of Anal Squamous Cell Carcinomas of Anal margin and Anal Canal [1]

**Fig. 2:**
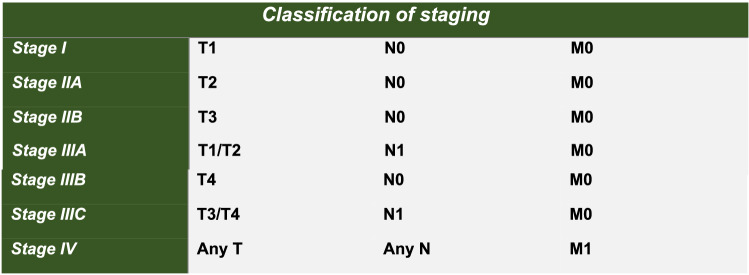
Classification of TMN staging 8th Edition for Anal Squamous Cell Carcinomas [1]

Adult patients (> 18 years) treated for a primary early-stage histologically confirmed ASCC between January 2000 and January 2020. Early-stage tumours are tumours that are defined as Stage 1, Stage 2A or Stage 2B by the 8th edition of the American Joint Committee on Cancer TMN staging [1] (see Figs. 1 and 2).

### Exclusion criteria

**Fig. 3:**
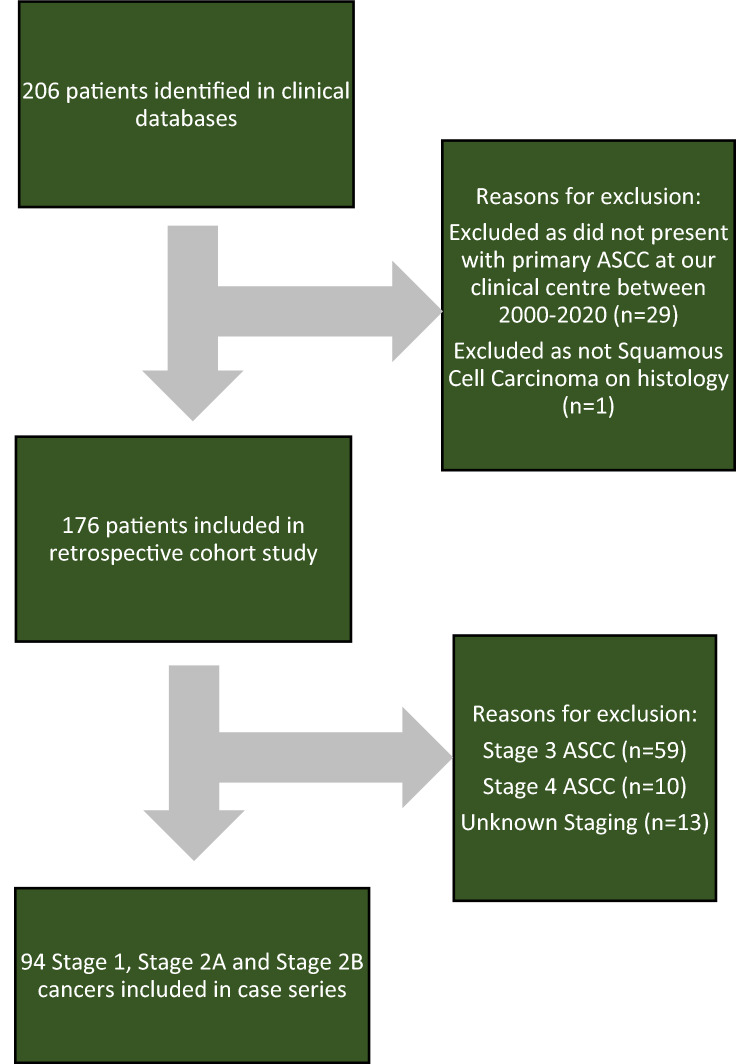
STROBE patient flowchart identifying reasons for patient inclusion and exclusion in retrospective cohort study and case series. *ASCC* anal squamous cell carcinoma

Patients under 18 years of age and patients that did not have their primary ASCC diagnosed at our clinical centre. Figure 3 shows data flow and reasons for exclusion from the study.

### Data collection

Data were retrieved from several prospectively maintained clinical databases at our clinical centre and combined to form a single data entry per patient. The data extracted included demographics, age, gender, co-morbidities including HIV status, previous HPV-related dysplasias and malignancies and immunosuppression. Staging, histopathological data and information about treatment received was also collected. PLWH and MSM were offered high-resolution aqnoscopy (HRA) and anal cytology screening by the HIV and sexual health physicians, screening outcomes were also included in the dataset. After treatment for ASCC, patients were followed-up for the first 5 years following the ACTII ASCC protocol [3]. After 5 years, PLWH and MSM re-entered the HRA and anal cytology screening programme. The end outcome measures for this study include recurrence, time to recurrence and 5-year disease-free survival.

### Statistical analysis

Data were analysed using SPSS Statistics software. ANOVA tests were used for comparison of means and Fisher’s exact tests were used for categorical variables. A statistically significant p value was defined as *p *< 0.05 for this analysis.

## Results

One hundred seventy-six patients with primary ASCC diagnosed between January 2000 and January 2020 were identified, within this cohort Ninety-four patients were classified as stage 1, stage 2A or stage 2B (see Figs. 1 and 2 for TMN staging classification) and were included in this study. The STROBE patient flowchart is shown in Fig. 3. Sixty-eight patients were male (72%) and 57 patients were PLWH (61%). Thirty-five patients (37%) had a prior diagnosis of high- or low-grade squamous intraepithelial lesions (LSIL) and 6 patients (6%) had a prior diagnosis of genitourinary intraepithelial neoplasia (GIN) (23% of women in the cohort). Twenty-five patients (27%) of patients in the cohort had well-differentiated tumours or malignant foci of ASCC only on histology.

There was no statistically significant difference between age, sex, HIV status, immunosuppression, history of HSIL or LSIL or GIN or tumour differentiation between the stage 1 and stage 2A/B subgroups (Table 1).

**Table 1:** Patient demographics and staging

Demographics	Stage 1 (*n *= 62)	Stage 2A (*n *= 27)	Stage 2B (*n *= 5)	*p* value
Age (years)	52.4	57.0	42.3	*p *= 0.064
Mean ± SD (range)	± 12.7 (32–90)	± 12.6 (39–80)	± = 9.4 (31–90)	
Sex	Male 47 (76%)	Male 17 (63%)	Male 4 (80%)	*p *= 0.487
	Female 15 (24%)	Female 10 (37%)	Female 1 (20%)	
HIV status	Positive 40 (65%)	Positive 13 (48%)	Positive 4 (80%)	*p *= 0.354
	Negative 11 (18%)	Negative 5 (19%)	Negative 1 (20%)	
	Not recorded 11 (18%)	Not recorded 9 (33%)	Not recorded 0 (0%)	
Other immunosuppression	3 (5%)	1 (4%)	0 (0%)	*p *= 1.000
Previous HSIL or LSIL	24 (39%)	9 (33%)	2 (40%)	*p *= 0.285
Previous GIN	6 (10%)	0 (0%)	0 (0%)	*p *= 0.259
Tumour differentiation				
Foci of ASCC	11 (18%)	2 (7%)	0 (0%)	
Well differentiated	10 (16%)	2 (7%)	0 (0%)	
Moderately differentiated	19 (31%)	9 (33%)	2 (40%)	
Poorly differentiated	6 (10%)	9 (33%)	1 (20%)	
Uncategorised	16 (26%)	5 (19%)	2 (40%)	*p *= 0.191

*HSIL* high-grade squamous intraepithelial lesions, *LSIL* low-grade squamous intraepithelial lesions, *HIV* human immunodeficiency virus, *GIN* genitourinary intraepithelial neoplasia, *ASCC* anal squamous cell carcinoma

Patients classified as stage 1 were less likely to receive chemoradiotherapy when compared to stage 2A and stage 2B (*p *< 0.000, Fisher’s exact test) and more likely to have local excision of tumour alone as the only treatment for their malignancy (*p *=  < 0.000, Fisher’s exact test). There was no statistically significant difference between staging groups in patients receiving radiotherapy only (Table 2). The use of chemoradiotherapy was associated with tumour differentiation (*p *= 0.003, Fishers exact test); indeed 93% of stage 1, 82% of stage 2A and 100% of stage 2B tumours that received chemoradiotherapy were either moderately or poorly differentiated.

**Table 2:** Treatment and staging

Treatment received	Stage 1 (*n *= 62)	Stage 2A (*n *= 27)	Stage 2B (*n *= 5)	*p* value
Chemoradiotherapy	19 (31%)	22 (82%)	4 (80%)	*p *< 0.000
Radiotherapy only	2 (3%)	0 (0.0%)	0 (0.0%)	*p *= 0.561
Local excision of tumour	45 (73%)	8 (30%)	1 (20%)	*p *< 0.001
Abdominoperineal resection (after recurrence)	1 (2%)	3 (11%)	0 (0%)	*p *= 0.119
Defunctioning stoma	2 (3%)	2 (7%)	1 (20%)	*p *= 0.172
Local excision of tumour only	34 (55%)	1 (4%)	0 (0%)	*p *< 0.000

Sixty-two patients (66%) had stage 1 disease. Forty-seven were male (76%) and 40 patients were PLWH (65%). Three (5%) had an immunosuppressive state other than HIV, 24 patients (39%) had a history of HSIL or LSIL and 6 (10%) had a previous diagnosis of GIN. Twenty-one (44%) stage 1 tumours were well differentiated or had a foci of malignancy on histopathology.

### Treatment of stage 1 tumours

Forty-five patients with stage 1 tumours underwent a local excision, 34 (76%) of them had local excision as the only treatment for their malignancy. With the exception of 1 patient who was classified as stage 2A, all patients who received a local excision as the only treatment for their malignancy were stage 1 at diagnosis. Stage 1 patients who had a local excision as their only treatment were younger than stage 1 patients that also received another treatment modality and this difference was statistically significant (*p *= 0.037, ANOVA) (Table 3). They also were more likely to have either foci of malignancy or well-differentiated tumours on histopathology (*p *= 0.002, Fisher’s exact test). Two patients with stage 1 tumours had a defunctioning stoma for symptom control prior to oncological treatment, they were both HIV positive and were early in the case series. One stage 1 patient who required an abdominoperineal resection after failure to respond to chemoradiotherapy had a long history of immunosuppression and steroid use. There was no statistically significant difference between stage 1 treatment groups when stratifying for sex, HIV status, immunosuppression or previous diagnosis of intraepithelial neoplasias.

**Table 3:** Treatment of stage 1 tumours

Demographics	Local excision only (*n *= 34)	Other treatment modality (*n *= 25)	*p* value
Age (years)	50.5	55.4	*p *= 0.037
mean ± SD, (range)	± 12.4 (32–81)	± 12.9 (33–90)	
Sex	Male 26 (76%)	Male 20 (80%)	*p *= 0.502
	Female 8 (24%)	Female 5 (20%)	
HIV status	Positive 22 (65%)	Positive 17 (68%)	*p *= 0.637
	Negative 5 (15%)	Negative 5 (20%)	
	Not recorded 7 (21%)	Not recorded 3 (12%)	
Other immunosuppression	0 (0%)	2 (8%)	*p *= 0.175
Previous HSIL or LSIL	14 (41%)	9 (36%)	*p *= 0.907
Previous GIN	4 (12%)	2 (8%)	*p *= 0.821
Tumour differentiation			
Foci of ASCC	8 (24%)	2 (10%)	
Well differentiated	8 (24%)	1 (12%)	
Moderately differentiated	6 (18%)	13 (39%)	
Poorly differentiated	1 (3%)	5 (10%)	
Not categorised	11 (32%)	4 (16%)	*p *= 0.002

*HSIL* high-grade squamous intraepithelial lesions, *LSIL* low-grade squamous intraepithelial lesions, *HIV* human immunodeficiency virus, *GIN* genitourinary intraepithelial neoplasia, *ASCC* anal squamous cell carcinoma

## Clinical outcomes

### Recurrence

Fifteen patients (16%) recurred on follow-up. There was no association with gender, HIV status, immunosuppressive states other than HIV and prior diagnosis of HPV-related dysplasias. However, patients who had a recurrence were older at ASCC diagnosis (mean age 55.4 years vs. 52.6 years, *p *= 0.006, ANOVA).

**Fig. 4:**
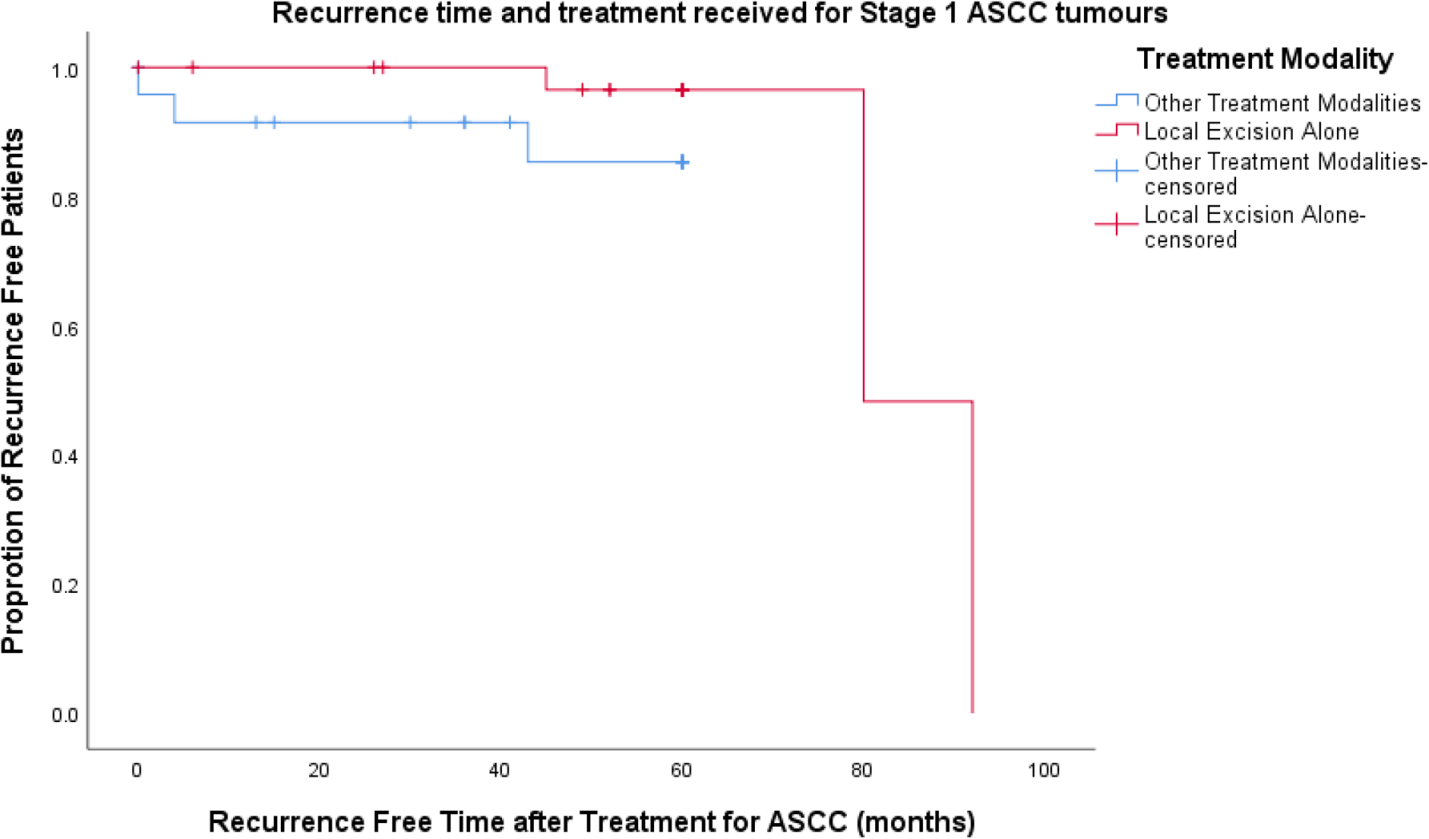
Kaplan–Meier curve demonstrating difference in time to recurrence in months between stage 1 treatment groups. *ASCC* anal squamous cell carcinoma

Eight patients who recurred were classified as stage 1 at ASCC diagnosis. There was no association between treatment received for stage 1 tumours and recurrence (Table 4), However, patients who had local excision as their only treatment had a longer time to recurrence (72.3 months vs, 27.3 months, *p *= 0.06, ANOVA) (Fig. 4).

**Table 4:** End outcomes of local excision alone compared to other treatment modalities

End outcomes	Local excision only (*n *= 34)	Other treatment modality (*n *= 54)	*p* value
Recurrence	3 (9%)	12 (22%)	*p *= 0.236
Time to recurrence, (months), mean ± SD	72.3 ± 24.4	27.3 ± 18.8	*p *= 0.06

**Fig. 5:**
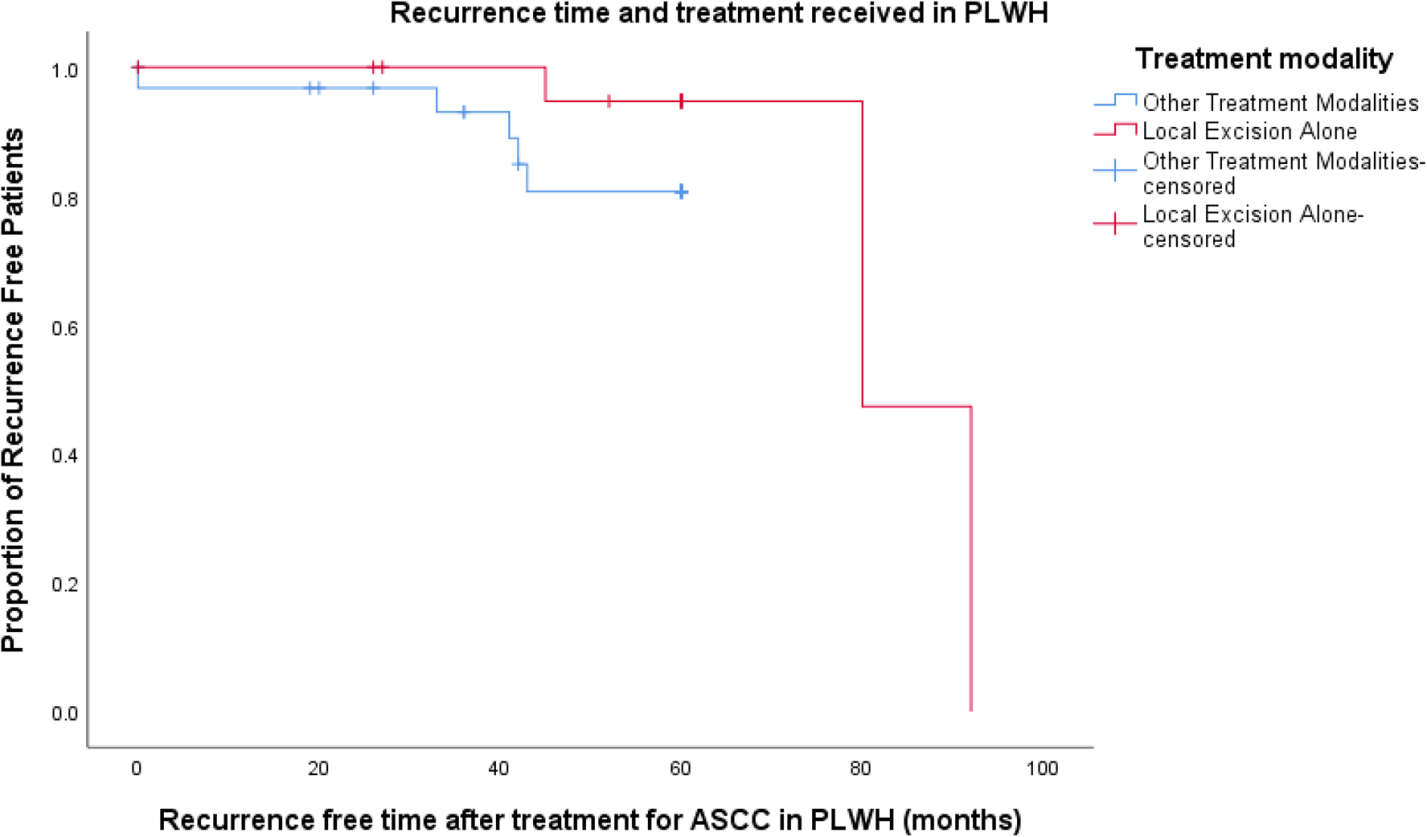
Kaplan–Meier curve demonstrating difference in recurrence time in months in PLWH when comparing patients who underwent local excision alone compared to other treatment modalities. *ASCC* anal squamous cell carcinoma, *PLWH* people living with human immunodeficiency virus

There was no statistical difference between age and previous HPV-related dysplasia between groups and no difference in 5-year disease-free survival and recurrence. However, like the case series as a whole; patients who underwent a local excision only for their malignancy were more likely have well differentiated or foci of malignancy on histological analysis (*p *= 0.029, Fisher’s exact test). Patients who had local excision alone also had recurrent disease later (*p *= 0.035, ANOVA) (Fig. 5). One patient with poorly differentiated stage 2A tumour had a local excision as their only treatment. This was a decision made by the patient and not a treatment plan recommended by the multidisciplinary team. The patient did not achieve 5-year disease-free survival.

Three stage 1 patients who had local excision as the only treatment for their malignancy eventually developed a recurrence at 45 months, 80 months and 92 months, respectively. All three were PLWH, presented again with T1/T2 node-negative tumours and have achieved good long-term outcomes to date after receiving chemoradiotherapy (Table 5).

**Table 5:** End outcomes local excision group compared to other treatment modalities in PLWH

End outcomes	Local excision only (*n *= 22)	Other treatment modality (*n *= 33)	*p* value
Recurrence	3 (14%)	7 (21%)	*p *= 0.812
Time to recurrence, (months), mean ± SD	72.3 ± 24.4	31.8 ± 18.2	*p *= 0.035

*PLWH* people living with human immunodeficiency virus

## Discussion

We undertook a case series review of the treatment of early-stage tumours extrapolated from a retrospective cohort examining the outcomes of the treatment of primary ASCC over 20 years in a HIV tertiary referral centre. Treatment choice was determined in specialist weekly multidisciplinary team meetings with colorectal surgeons, radiologists and oncologists present.

There were three separate analyses included in this case series; the first being the overall demographics and outcomes of all patients treated for stage 1, 2A and 2B disease between January 2000 and January 2020, the second a sub-analysis of stage 1 tumours stratified by patient treatment and the third a sub-analysis of early-stage tumours in PLWH only.

The interest in organ sparing treatment for ASCC stems from the Lower Anogenital Squamous Terminology (LAST) criteria which identified a new category of ASCC; “superficially invasive squamous cell carcinoma” (SISCCA) that may be amenable to local excision alone [25].

As a result, some clinical guidelines now recommend local excision alone in T1N0 well-differentiated tumours with a clear margin.[9, 10, 13, 26]. This is based on several retrospective studies being able to demonstrate no difference in long-term outcomes for patients with stage 1 tumours that are treated with local excision alone when compared to stage 1 patients that receive chemoradiotherapy [17–22].

Clinical guidelines also recommend that all patients considering local excision alone should be discussed in an expert ASCC multidisciplinary team meeting. We believe this is increasingly important since our results demonstrate that recurrence in PLWH can occur after the classic 5-year follow-up period. An expert ASCC multidisciplinary team meeting should by definition have access to a functioning screening programme for surveillance after treatment. This is not just for identifying ASCC but also as studies have shown that a high number of patients have persistence of high-grade anal intraepithelial neoplasia after local excision [27].

Currently, surveillance programmes are not widespread or are they recommended by clinical guidelines as there is no evidence base that surveillance after treatment for ASCC is beneficial. This can leave patients requiring long-term follow-up for ASCC in a difficult situation as high-resolution anoscopy (HRA), the gold standard for detection of anal HSIL detection, is only recommended when used by expert practitioners with high volumes of patients, therefore, the availability of expert practitioners to perform HRA for long-term follow-up of patients treated for ASCC can be scarce [9]. Societies such as the International Anal Neoplasia Society (IANS) are trying to rapidly train practitioners to be able to provide this service effectively worldwide but until guidance changes all patients that are being considered for a local excision alone should be considered for referral to a clinical centre with an expert multidisciplinary team that has access to an appropriate long-term follow-up programme.

We believe that the patients most likely to benefit from organ sparing treatment are patients with risk factors on surveillance programmes, the most common being PLWH who are also MSM. Research available from other clinical centres has not previously investigated the outcomes of PLWH after local excision. The recommendations in the clinical guidelines regarding the viability of PLWH ha ving local excision alone are based on a previous case series published at our clinical centre in 2016, which was only able to include 15 patients with a median follow-up time of 4 years [23]. We hope that this update from our centre will help to guide recommendations in the future whilst we wait for the results from the ACT3 arm of the PLATO trial [16].

### Strengths and limitations

This is an update of the long-term outcomes of PLWH and local excision from our HIV tertiary referral clinical centre with a surveillance programme available for high-risk patients. This also is the only case series that compares the long-term outcomes of HIV negative and PLWH with early cancers treated with local excision alone. Our data were extracted from multiple prospective clinical databases and taken from a 20-year retrospective cohort study that has recently published its clinical outcomes [15]. Our case series is the only study available in the literature that is able to demonstrate that HIV status does not adversely affect the outcomes of local excision in stage 1 tumours. Indeed, PLWH, if they do develop recurrence, appear to do so later than HIV-negative patients, suggesting that for PLWH a watchful waiting approach can be beneficial assuming they have adequate expert long-term follow-up.

As with any retrospective study design, our recommendations are potentially limited by recall bias and the quality of the information documented.

Unfortunately, we were not able stratify patients by their sexual history and practices as this was rarely documented in the notes, especially earlier in the cohort study when stigma was more of a concern. We would have liked to have been able to evaluate the outcomes of PLWH MSM separately as we believe this is the group that could potentially benefit most from local excision alone. More research needs to be undertaken to evaluate the acceptability of organ sparing treatment in this patient subgroup.

Our historical histopathology records predate the term SISCCA and the available reports only state whether a tumour was fully excised. We were, therefore, unable to complete a sub-analysis of tumours that were classified as SISCCA alone. Similarly, we were unable to stratify location of tumour in the complete analysis. Except for 1 patient with stage 2A disease discussed earlier, all patients who underwent local excision as their only treatment for malignancy were stage 1 and had a complete excision of a tumour located within the anal verge. The documentation for patients given further treatment was less specific so it was not possible to accurately describe how many patients who received chemoradiotherapy had anal verge or anal canal tumours. A significant proportion of PLWH with foci of malignancy were identified incidentally after a presumed benign procedure such as a haemorrhoidectomy, the documentation of the location of malignancy in this circumstance is, understandably, less thorough as a serious pathology was not suspected at the time of the operation. This limited our ability to be able to describe whether a recurrence is a true recurrence of a treated tumour or a novel malignancy in the perianal area especially in PLWH who had a long time to recurrence.

Further research needed includes quality of life outcomes after organ sparing treatment in patents with ASCC.

## Conclusions

In our experience, local excision alone and close follow-up is an acceptable management plan for stage 1 ASCC with advantageous histology regardless of HIV status. PLWH had late recurrence, therefore, we would recommend they enter a long-term HRA screening programme provided by expert practitioners once 5-year follow-up is completed.

## Data Availability

The complete datasets generated and analysed in this study are not publicly available without ethical approval as they contain personally identifiable data from hospital databases. Anonymised supplementary information can be requested from the corresponding author if the request is reasonable and meets the conditions of the ethical approval granted to undertake this study.
